# Super-resolution microscopy reveals significant impact of M2e-specific monoclonal antibodies on influenza A virus filament formation at the host cell surface

**DOI:** 10.1038/s41598-019-41023-5

**Published:** 2019-03-14

**Authors:** Annasaheb Kolpe, Maria Arista-Romero, Bert Schepens, Silvia Pujals, Xavier Saelens, Lorenzo Albertazzi

**Affiliations:** 1 VIB-UGent Center for Medical Biotechnology, Technologiepark-Zwijnaarde 71, Ghent, B-9052 Belgium; 2 Department of Biomedical Molecular Biology, Ghent University, Ghent, B-9052 Belgium; 3 Nanoscopy for Nanomedicine Group, Institute for Bioengineering of Catalonia (IBEC), C\Baldiri Reixac 15-21, Helix Building, 08028 Barcelona, Spain; 4 Department of Biomedical Engineering, Institute for Complex Molecular Systems (ICMS), Eindhoven University of Technology, 5612AZ Eindhoven, The Netherlands

## Abstract

Influenza A virions are highly pleomorphic, exhibiting either spherical or filamentous morphology. The influenza A virus strain A/Udorn/72 (H3N2) produces copious amounts of long filaments on the surface of infected cells where matrix protein 1 (M1) and 2 (M2) play a key role in virus filament formation. Previously, it was shown that an anti-M2 ectodomain (M2e) antibody could inhibit A/Udorn/72 virus filament formation. However, the study of these structures is limited by their small size and complex structure. Here, we show that M2e-specific IgG1 and IgG2a mouse monoclonal antibodies can reduce influenza A/Udorn/72 virus plaque growth and infectivity *in vitro*. Using Immuno-staining combined with super-resolution microscopy that allows us to study structures beyond the diffraction limit, we report that M2 is localized at the base of viral filaments that emerge from the membrane of infected cells. Filament formation was inhibited by treatment of A/Udorn/72 infected cells with M2e-specific IgG2a and IgG1 monoclonal antibodies and resulted in fragmentation of pre-existing filaments. We conclude that M2e-specific IgGs can reduce filamentous influenza A virus replication *in vitro* and suggest that *in vitro* inhibition of A/Udorn/72 virus replication by M2e-specific antibodies correlates with the inhibition of filament formation on the surface of infected cells.

## Introduction

Influenza A viruses are enveloped with a negative-sense RNA genome consisting of eight ribonucleoprotein segments. These eight genome segments encode for at least 11 viral proteins, including the membrane proteins hemagglutinin (HA), neuraminidase (NA), and the proton-selective ion channel matrix protein 2 (M2). The M2 protein fulfills important functions during virus entry and is also involved in virus assembly^1–3^. Influenza virions are released from infected cells by budding, a process that occurs in the so-called budozone in the plasma membrane, where the viral hemagglutinin (HA) and neuraminidase (NA) accumulate. M2 resides at the periphery of the budozone, where it plays an important role during virion assembly and budding by associating with M1 and inducing membrane curvature^1,4,5^. The recent reports showed that NA and HA might not enriched with cholesterol and sphingolipid^6,7^.

Influenza virus budding results in the formation of filamentous, bacilliform or spherical particles, depending on the virus strains that are used. Infection with A/WSN/33 (H1N1), for example, predominantly gives rise to spherical virions, whereas infection of cells with the strain A/Udorn/72 (H3N2) produces a mixture of spherical and filamentous virions^2,8–10^. Filamentous influenza virions are thought to be the predominant form in the upper respiratory tract of influenza patients^8,11,12^ and were also detected in 2009 H1N1 pandemic virus isolates^13^. Indeed, the general view is that primary human influenza virus isolates are filamentous in appearance, but convert into predominantly spherical virions after serial passage in embryonated chicken eggs^14^. Spherical and filamentous virus particles are equally infectious *in vitro*^8,10,15^. Recent experimental studies in animal transmission models have shown that filamentous virus particles formation correlates with virus transmissibility between co-housed guinea pigs and ferrets^16,17^.

Previous studies have shown that influenza A virus filament formation is a genetic trait that maps to the M1 coding information^10,18,19^. Transfer of the A/Udorn/72 M1 protein coding sequence into the A/WSN/H1N1 genetic background allows filament formation, whereas substitution of specific A/WSN/H1N1 M1 residues in the A/Udorn/72 genetic background results in the production of spherical virions^19,20^. Furthermore, additional studies have suggested that the M2 protein may also be involved in filament formation^1,8,21^. In particular an amphipathic alpha helix in the cytoplasmic part of M2 has been proposed to contribute to the formation and stability of filamentous virion formation^8^.

Antibodies directed against M2e (the extracellular part of M2), can protect against experimental influenza A virus challenge *in vivo* by an Fcγ Receptor-dependent mechanism^22,23^. Some influenza A virus strains, however, are also susceptible to a direct *in vitro* antiviral effect of M2e-specific IgGs^24^. In this case, M2e-specific IgGs perturb critical interactions between the M1 and M2 proteins, which in turn affect the interaction of M1 with the viral ribonucleoprotein complexes. As a consequence, virions assembly is compromised^25^. Evidence for such an effect on the interaction between M1 and M2 is based on the observation that treatment of influenza A virus-infected cells with the M2e-specific monoclonal antibody (MAb) 14C2 results in a loss of filament formation and reduces infectivity of some influenza A virus strains such as A/Udorn/72 *in vitro*^8,25^. M2e-specific antibody-mediated fragmentation of filamentous virions appears to be due to the induction of a conformational change in the M2 protein, which alters membrane curvature^1,8^. Treatment of infected cells with M2e-specific MAb 14C2 antibody was also shown to inhibit viral assembly and release^26^. Several A/Udorn/72 virus variants that are resistant to the inhibitory effects of 14C2 MAb have mutations within viral RNA segment 7, which codes for the M1 and M2 proteins^27^. The M2 protein does not associate with rafts despite possessing a cholesterol recognition/interaction amino acid consensus (CRAC) domain, which has been shown in other proteins to mediate cholesterol binding^28^. The CRAC domain in M2 perhaps provides an affinity for cholesterol-rich regions of the budozone to ease the scission event during the viral budding process^29,30^. However, the notion that influenza HA associates with lipid-raft domains has been challenged recently^6,31^.

Due to the small size of the influenza particles (approximately 100 nm in width) and the filaments formed on infected cells, characterization of these structures is almost impossible using conventional fluorescence microscopy techniques such as confocal microscopy, because the diffraction barrier of microscopic techniques does not allow resolving structures smaller than 250 nm. Therefore, studies of the budding process of influenza viruses have been performed using electron microscopy (EM) sometimes combined with immuno-gold staining of viral proteins^32^. However, EM has several limitations to study biological samples. The preparation of the sample for EM is complex and time consuming and requires expensive and sensitive materials.

To overcome those limitations and facilitate the acquisition of precise images of budding influenza virions, we used super-resolution microscopy (SRM), in concrete stochastical optical reconstruction microscopy (STORM), a technique that allows the location of molecules to be determined with nanometer-scale precision at a very high resolution^33,34^ and although so far has been used to study the cell biology of influenza viruses^34–38^, it has never been used to study the role of M2 in filament formation and structures. STORM is a nanoscopy technique that allows to obtain images with a resolution of approximately 20 nm^39^. STORM takes advantage of sequential photoswitching of certain dyes. This relies on the stochastic process of a fluorophore (e.g. attached to an antibody specific for the structure of interest) to be repeatedly turned on and off by a chemical reaction, while images are sequentially acquisitioned. This allows to acquire all fluorophores of the sample individually accumulating sequential images during the photoswitching. By determining the position of each fluorophore individually with high accuracy using a Gaussian fitting allows to reconstruct a high resolution image^40^. This method is compatible with immunostaining allowing to obtain new insights at a nanoscale level of assembling viral structural proteins, for example at the budozone.

In the present study, we have investigated the effect of three M2e-specific MAbs on influenza A virus plaque formation and infectivity *in vitro*. We have utilized confocal and super-resolution STORM microscopy to characterize filaments formation that is associated with influenza A/Udorn/72 replication. We further investigated M2e-specific antibody-induced perturbation of filament formation and fragmentation of pre-existing filaments in influenza A/Udorn/72 infected cells.

## Results

### M2e-specific MAbs reduce plaque growth and infectivity of influenza A/Udorn/72 virus *in vitro*

**Figure 1:**
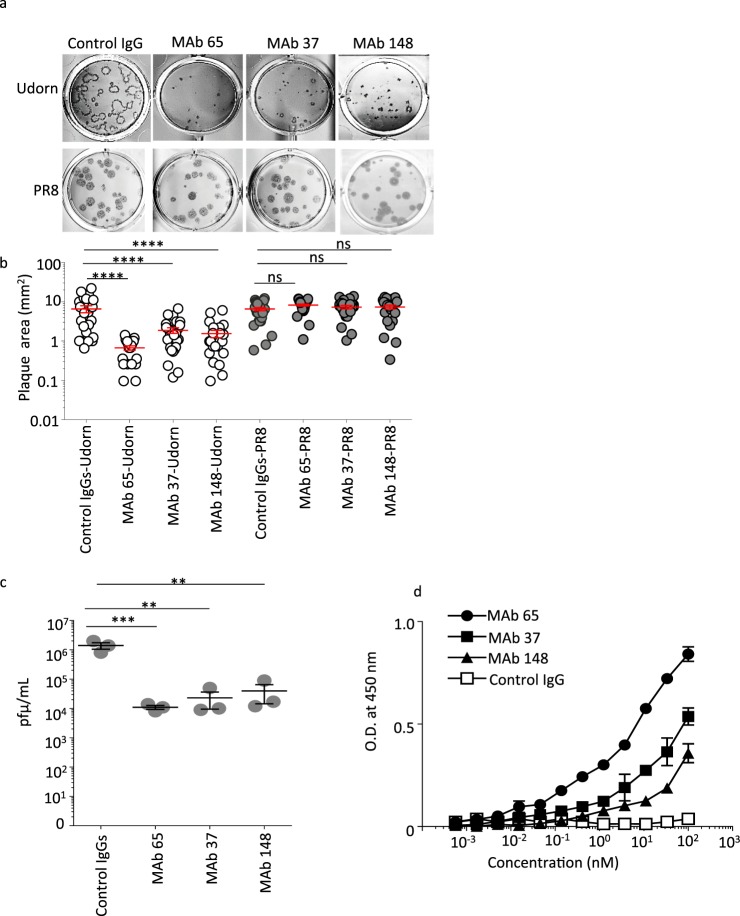
M2e-specific IgGs inhibits plaque growth and infectivity of A/Udorn/72 *in vitro*. (**a**) MDCK cells were infected with A/Udorn/72 or PR8 virus at 20–50 PFU/well and subsequently overlaid with 1.2% Avicel in medium containing M2e-specific IgGs (MAbs 37, 65, 148) or isotype control IgG (IgG1 + IgG2a) at 100 μg/mL and 2 μg/mL TPCK-treated trypsin. After 72 h of incubation at 37 °C, the cells were fixed with 4% paraformaldehyde, permeabilized with 1% Triton X-100 and the plaques were visualized by staining with influenza polyclonal goat anti-influenza ribonucleoprotein (RNP). **(b)** Quantification of the plaque size based on ImageJ analysis. The graph shows the area of each plaque and the bars represent the mean area ± SEM. The Udorn plaque size of all three M2-specific IgGs (MAbs 37, 65, 148) treated cells is significantly smaller than the plaque size of control IgG treated cells (One-way ANOVA). **(c)** MDCK cells were treated with M2e specific MAbs (65, 37, and 148) or Control IgG at 100 μg/ml and infected with A/Udorn/72 at an MOI of 0.01 in TPCK-treated trypsin containing medium. Twenty four hours after infection, the medium was harvested and titrated by plaque assay (**d)** MDCK cells were infected with A/Udorn/72 virus. Twenty-four hours later, the cells were incubated with a dilution series of MAb 37, MAb 65, MAb 148 or control IgG, followed by fixation with 4% paraformaldehyde and detection by a cellular ELISA. Non-significant (ns), **p ≤ 0.01, ***p ≤ 0.001; ****p ≤ 0.0001.

The M2-specific mouse IgG1 MAb 14C2 was previously shown to be able to inhibit influenza A/Udorn/72 virus plaque growth and infectivity *in vitro*^8,24,26^. In line with this, we demonstrate that the M2e-specific IgG MAbs 37 (IgG1) and 65 (IgG2a), both directed against a similar part of M2e (encompassing residues Thr4-Trp15), and MAb 148 (IgG1, directed against Ser2-Thr9) can reduce the plaque growth of A/Udorn/72 virus (Table 1, Fig. 1a).

**Table 1:** Monoclonal antibodies used in the study.

Name of MAb	Epitope specificity	Isotype	References
MAb 37	Influenza M2e	IgG1	^55^.
MAb 65	Influenza M2e	IgG2a	^55, 57^
MAb 148	Influenza M2e	IgG1	^57^
Control MAb	Hepatitis B core	IgG1	^55^
Control MAb	Respiratory syncytial virus small hydrophobic protein	IgG2a	^55^

The plaque size of Udorn was significantly smaller in the presence of all three M2e-specifc MAbs used at a concentration of 100 µg/mL compared to isotype control antibody (Fig. 1a,b). In contrast, the plaque growth of PR8 virus was not affected by M2e-specific MAbs 65, 37, 148. (Fig. 1a,b). In a multicycle growth setup, all three M2e-specific MAbs significantly reduced the amount of newly produced infectious A/Udorn/72 virus (Fig. 1c). The M2e-specific MAbs 65, 37, and 148 bound to M2 expressed on the surface of A/Udorn/1972 (H3N2) virus-infected cells with estimated Kds of 2.073 nM, 6.957 nM, and 14.82 nM, respectively, on the basis of a cellular ELISA (Fig. 1d). Thus, M2e-specific MAbs bind to M2 expressed on the surface of A/Udorn/1972 (H3N2) virus-infected cells and can reduce infectivity and plaque growth of A/Udorn/72 *in vitro*.

### Confocal and STORM imaging of A/PR8/H1N1 and A/Udorn/H3N2 infected cells

**Figure 2:**
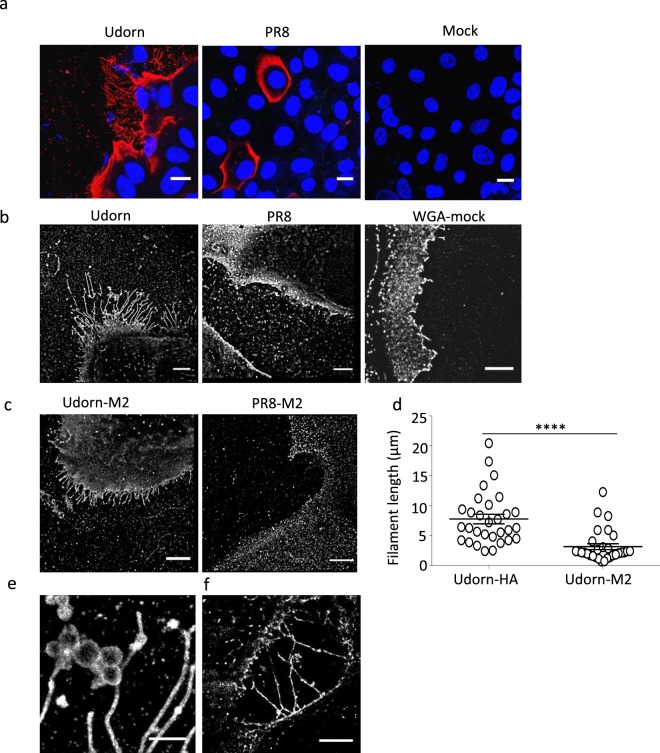
STORM imaging of A/Udorn/72/H3N2 infected MDCK cells reveals filaments with Archetti bodies. MDCK cells were seeded in 8 well microslides, infected at an MOI of 5 PFU/cell with influenza A/Udorn/72 or A/PR8/H1N1 virus for 24 h, fixed, stained for HA (using convalescent mouse serum against A/Udorn/72 or A/PR8/H1N1) and M2 separately. **(a)** Confocal images of filaments on infected cells, immuno-labeled for HA (red) and DAPI (Blue). Scale bar = 10 μm. STORM images of filaments on A/Udorn/72 virus infected cells, immuno-labeled for HA **(b)** and M2 **(c) Scale bar 5 μm**. **(d)** HA-containing filaments are significantly longer than M2-containing filaments (n = 30). **(e)** STORM images of filaments on A/Udorn/72 virus infected cells, immuno-labeled for HA showing Archetti bodies. Bar 1 μm **(f)** STORM images of filaments on A/Udorn/72 virus infected cells, immuno-labeled for HA showing HA-containing protrusions making connections to neighboring cells. Bar 5 μm. The experiments were performed in triplicate wells for each condition and repeated at least three times with similar results. ****p ≤ 0.0001.

Previously it has been shown that immunostaining and light microscopy can be used to visualize filamentous structures that emerged from the surface of the infected cells^8,41,42^. Here we used confocal and super-resolution STORM microscopy to characterize filament formation on the surface of A/Udorn/72 infected cells. By combining these two microscopy techniques, we wanted to gain a detailed insight in the morphology of those filaments and to understand the effect of a set of well-characterized M2e-specific MAbs on A/Udorn/72 filament formation. Confocal and STORM imaging of MDCK cells fixed and immuno-stained 24 h after infection revealed filamentous structures that emerged from the surface of the infected cells, whereas infection with PR8 virus did not result in the formation of such structures (Fig. 2a,b). STORM analysis of uninfected MDCK cells showed few small filopodia when stained with wheat germ agglutinin (WGA-mock) (Fig. 2b). HA is the most abundant membrane protein in influenza A virions, NA is approximately 10-fold less abundant and M2 is scarcely represented^43,44^. In the plasma membrane of infected cells, however, M2 is expressed at high levels^8,24,45,46^. Given the involvement of M2 in filament formation (budding virions), we investigated if M2 is detectable at the sites of budding filaments on infected cells. For this purpose, influenza A/Udorn/72 virus-infected MDCK cells were stained with polyclonal convalescent mouse serum or M2e-specific IgG1 MAb 37 at 24 h after infection and then analyzed by STORM. HA and M2 were both present in A/Udorn/72 filaments using super-resolution STORM microscopy (Fig. 2b,c). Staining of the infected cells with a convalescent mouse polyclonal A/Udorn/72 serum revealed filaments with a length of 2–20 µm length and a width of 80–250 nm (Fig. 2b). Filaments can be traced to their origin on the cell surface using super-resolution STORM imaging, where M2 was observed mainly in the basilar part of budding filaments in A/Udorn/72 infected cells (Fig. 2c). The length of A/Udorn/72 HA-containing filaments was significantly longer than the M2 positive filaments (n = 30) (Fig. 2d).

The super-resolution STORM analysis revealed different filament structures, including branched filaments and filaments with enlarged oval/round structures (Fig. 2e). The latter structures have an apparent width of 230 to 663 nm and have been described previously as Archetti bodies^9,34,47^. It has previously also been reported that the A/Udorn/72 virus can spread to neighboring cells by using intracellular connections^48^. The STORM analysis showed that, long filaments or long HA-containing protrusions connected neighboring cells (Fig. 2f). Taken together, super-resolution STORM microscopy proved to be a useful tool to study the detailed structure and protein distribution in filaments that emerge from influenza A virus infected cells.

### M2e-specific IgGs suppress filament formation and cause fragmentation of pre-existing filaments

**Figure 3:**
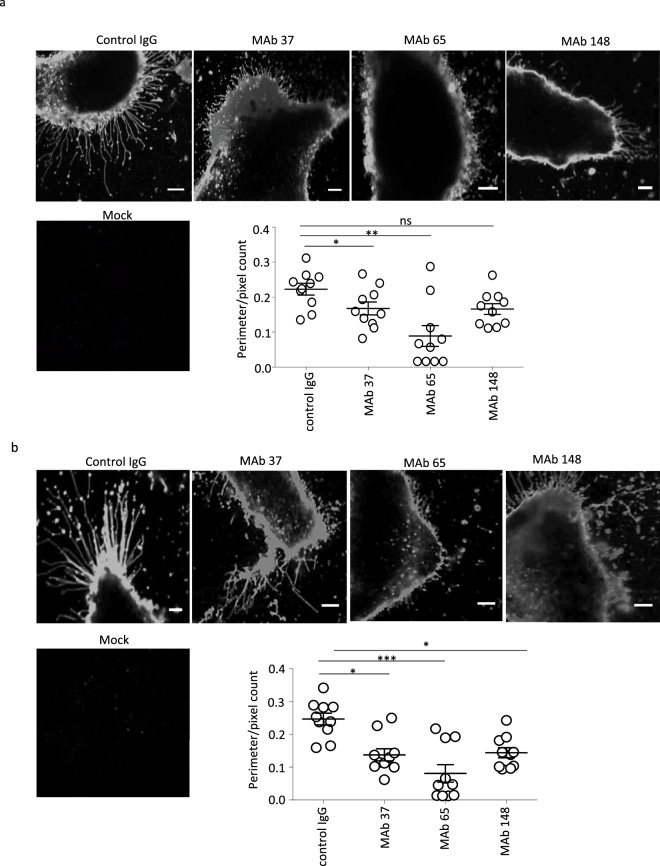
Confocal imaging reveals significant impact of M2e-specific monoclonal antibodies on the filament morphology of influenza A/Udorn/301/72 (H3N2) virus infected cells. MDCK cells were seeded in 8 well microslides, treated with M2e-speficic MAb 37 (IgG1), MAb 65 (IgG2a), MAb 148 (IgG1), or isotype control IgG1 + IgG2a at 20 μg/mL at 0 h or 24 h post infection with A/Udorn/72 at MOI 5 in serum-free medium. A mock infected control was included. The cells were then washed with PBS and fixed with 2% PFA at room temperature for 20 min. Infected cells were visualized by immune-staining with polyclonal convalescent mouse serum directed against A/Udorn/72, followed by Alexa Fluor 647 Donkey Anti-Mouse IgG serum and confocal imaging using Zeiss LSM 780 confocal microscope (Carl Zeiss, Germany) with 40x magnification. (**a**) Confocal images showing loss of filaments when MDCK cells are treated with M2e-speficic MAbs at 0 h post infection. (**b**) Confocal images showing fragmentation of pre-existing filaments when MDCK cells are cells treated with M2e-speficic MAbs at 24 h post infection. For confocal image analysis, the ratio of perimeter to the surface of cells analysis was performed in Volocity imaging software (Perkin Elmer). Scale bar = 5 μm. Perimeter/pixel count ratio is significantly lower in M2e-specific MAb treated cells than isotype control IgG treated cells. The experiments were performed in triplicate wells for each condition and repeated at least three times with similar results. One-way ANOVA with multiple comparisons correction (Kruskal–Wallis test). non-significant (ns), *p > 0.05, * p ≤ 0.05, ** p ≤ 0.01.

**Figure 4:**
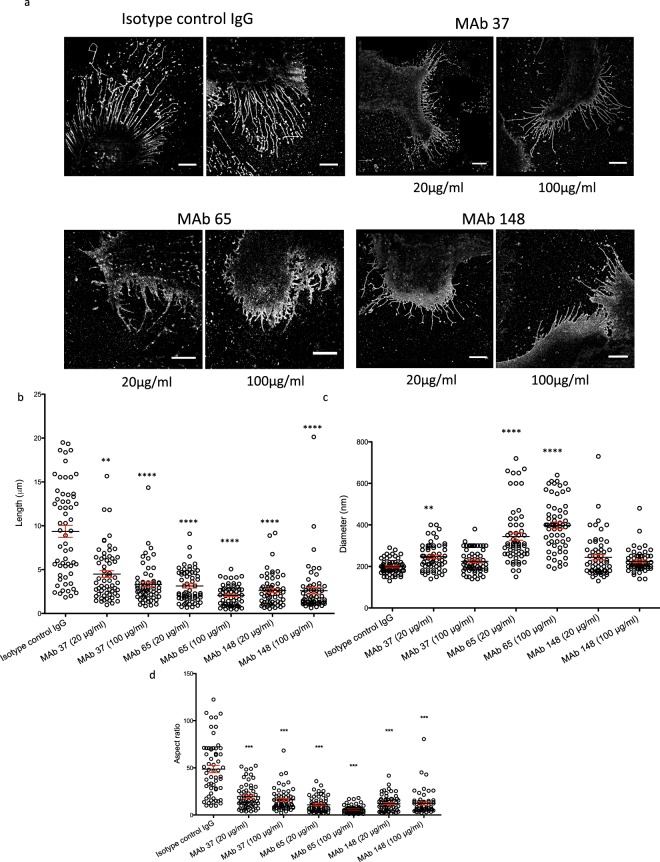
Super-resolution microscopy analysis shows inhibition of filament formation by M2e-specific IgGs (n = 60). MDCK cells were seeded in 8 well microslides, treated with M2e-speficic MAb 37 (IgG1), MAb 65 (IgG2a), MAb 148 (IgG1), or isotype control IgG1 + IgG2a at 20 or 100 μg/mL and then infected with A/Udorn/72 at MOI 5 and incubated for 24 Hrs at 37 °C in serum-free medium. The cells were then washed with PBS and fixed with 2% PFA at room temperature for 20 min. Infected cells and A/Udorn/72 filaments were visualized by immune-staining with polyclonal convalescent mouse serum directed against A/Udorn/72, followed by Alexa Fluor 647 Donkey Anti-Mouse IgG serum **(a)** STORM images of MDCK cells infected with influenza and treated with different MAb at different concentrations (20 µg/ml and 100 µg/ml). **(b)** Representation of the length (µm) of all filaments quantified (n = 60) from infected cells and treated with MAb. **(c)** Representation of width (diameter (nm)) of filaments quantified (n = 60). (**d**) Representation of the aspect ratio (length/diameter) of all filaments quantified (n = 60). Bar = 5 µm. The length of A/Udorn/72 filaments was significantly reduced in M2e-specific MAb treated cells (B). The experiments were performed in triplicate wells for each condition and repeated at least three times with similar results. One-way ANOVA. Non-significant (ns), **p ≤ 0.01; ***p ≤ 0.001; ****p ≤ 0.0001.

Despite multiple passages in cell culture, the influenza A/Udorn/72 virus strain has retained the capacity to produce filamentous virus particles. The M2-specific antibody 14C2 can restrict *in vitro* growth and assembly of the A/Udorn/72 virus, prevent filament formation, and cause the fragmentation of pre-existing filaments. Inhibition of the M2 ion channel function with amantadine, however, does not affect filament formation by A/Udorn/72 infected cells, whereas this drug prevents the post-entry fragmentation of filamentous virions in the endosomes^2,8,24–26^. In order to know whether our M2e-specific IgGs can also perturb filament formation, we treated A/Udorn/72 infected cells with MAbs 65, 37, 148 or control IgG at concentrations of 20 or 100 µg/mL and analyzed the outcome by confocal (shown in Fig. 3) and STORM (shown in Fig. 4) imaging.

Numerous long filaments were observed at cell surfaces when A/Udorn/72 virus-infected cells were examined by confocal and super-resolution STORM microscopy in isotype control IgG treated samples (Figs 3a and 4a). The filaments seen by confocal and STORM imaging may or may not correspond to the previously described long filamentous virus particles observed during budding at the cell surface of A/Udorn/72 infected cells^26^. In contrast, cells incubated with any of the M2e-specific MAbs 65, 37, 148 displayed much shorter filaments that contained the major viral spike proteins (Figs 3a and 4a). For confocal analysis, the ratio of the perimeter of a cell to the surface of that cell was used as a parameter to study the level of filament formation on cells. The perimeter/pixel count ratio was significantly lower in M2e-specific MAb 37 and MAb 65 treated cells, confirming that inhibition of filament formation by M2e-specific MAbs (Fig. 3a). Thanks to the STORM images we can study and see how the shape of the filaments changed strongly compared to the negative control. In all samples treated with MAb filaments showed a strong different phenotype compared to the negative control filaments. The most significant change was noticed with MAb 65 treated cells but not exclusively. Filaments formed after the incubation of MAbs displayed a very different filament profile compared to the control antibody treatment: the filaments were shorted, broken and wider (Fig. 4a). Also in some cases (Fig. 4a MAb 65) filaments are completely defective and don’t look like filament at all, but protrusions with a triangle shape.

In addition, these structures were not protruding in parallel but they were curved and produced branches along the filament. Most new filaments appear weaker and easier to break, whereas filaments from negative control are long and not broken at all.

Since the changes of the phenotype in the filaments after the incubation with the M2e-specific MAbs were very clear, we decided to quantify these changes by measuring the width and length of the filaments, the two most remarkable features. To ensure a correct measurement, we developed a criterion for selecting the filaments to be counted in order to have a representative collection of samples: the filaments selected should have the same width along the filament, be intact (not broken), lack branches and the structure must be fully reconstructed. For this purpose we analyzed cells that were infected at 5 MOI and we tested two different concentrations (100 and 20 µg/ml) of the M2e-specific MAbs (Fig. 4b).

We counted in total 60 filaments from 7–9 different cells per condition with an average of 8 filaments per cell. To study the distribution of all filaments per condition, we plotted the full length and width of all filaments measured individually (n = 60).

The length of the filaments of the negative control showed a wide size distribution (Supplementary Information 2, Supplementary Table 1), ranging between 2 and 20 µm. When the M2e-specific MAbs were applied at 20 µg/ml, we observed a decrease in length and an increase of the homogeneity of all samples (between 0.5 and 10 µm) (Fig. 4b). Increasing the concentration of the MAbs to 100 µg/ml produced smaller filaments with similar sizes and the samples were even more homogeneous (Fig. 4b, Supplementary Information 2).

Performing an ANOVA test on this data, we can see how these differences between al populations of filaments treated with MAbs in comparison to the negative control are statistically significant in all cases. Also the variance of these populations are much smaller than in the negative control (Supplementary Table 1). Thus, treatment of A/Udorn/72 virus infected cells with the M2-MAbs produced a more homogeneous population of filaments that, furthermore, were much shorter.

The width is the second feature that was measured (Fig. 4c). The negative control showed a homogeneous width of the filaments of around 170 nm. Cells treated with MAb 65 produced filaments with variable widths, where the average width was around 250 nm and ranged from 100 nm to 610 nm as deduced from the STORM imaging (Fig. 4c, Supplementary Information 2). The distribution of width of these filaments was plotted, showing an increase of distribution of diameter when cells are treated with MAb 65 (both concentrations) and MAb 37 (20 µg/ml). The ANOVA test indicated a statistically significant difference between the population of filaments in the negative control and MAb 65.

Incubation of infected cells with MAb 148 presented with a small increase in the heterogeneity of the filaments. Similar results were obtain with the incubation of MAb 37 at high concentrations (100 µg/ml). The width was not dramatically changed by the incubation with these two MAbs (Fig. 4c).

To finalize, we also measured the aspect ratio (length/diameter) of each filament and obtained the average of all conditions (Fig. 4d, Supplementary Table 1). The aspect ratio decreased dramatically with the treatment, being also almost 9 times smaller in filaments with MAb 65 (100 µg/ml) than the aspect ratio in the negative control. In the rest of conditions the aspect ratio is 4 times smaller than in the negative control. With this information we can notice that cells treated with MAb 65 produced shorter and wider filaments, as previously quantified separately.

We observed copious amounts of filaments when cells are infected with high MOI (MOI 5) of A/Udorn/72 virus. However, M2e-specific antibody mediated inhibition of filament formation was also seen in MDCK cells that were infected at a low MOI (Supplementary Figure 1: 30, 90, 300 pfu/30,000 cells). Notably, these are sub-diffraction features that were not accessible quantitatively with confocal microscopy.

**Figure 5:**
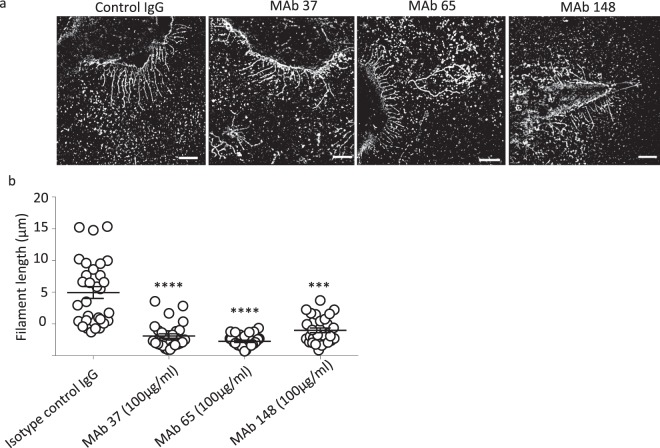
Super-resolution microscopy analysis shows that M2e-specific IgGs can disrupt pre-existing filaments (n = 50). MDCK cells were seeded in 8 well microslides, treated with M2e-speficic MAb 37 (IgG1), MAb 65 (IgG2a), MAb 148 (IgG1), or isotype control IgG1 + IgG2a at 20 μg/mL at 24 h post infection with A/Udorn/72 at MOI 5 in serum-free medium. The infected cells were incubated with M2e-specific MAbs for 1 h at 37 °C. The cells were then washed with PBS and fixed with 2% PFA at room temperature for 20 min. infected cells and A/Udorn/72 filaments were visualized by immune-staining with polyclonal convalescent mouse serum directed against A/Udorn/72, followed by Alexa Fluor 647 Donkey Anti-Mouse IgG serum. **(a)** STORM images showing disruption pre-existing filament when MDCK cells are cells treated with M2e-speficic MAbs at 24 h post infection. **(b)** Representation of the length (µm) of all filaments quantified (n = 50) from infected cells and treated with MAb. Bar = 5 µm. The experiments were performed in triplicate wells for each condition and repeated at least three times with similar results. One-way ANOVA with multiple comparisons correction (Kruskal–Wallis test). ***p ≤ 0.001; ****p ≤ 0.0001.

Importantly, infected cells were incubated with M2e specific MAbs 65, 37, 148 throughout infection cycle (24 HPI), therefore the above data do not allow us to distinguish whether M2e specific MAbs treatment blocks the formation of filamentous particles or has a direct effect on pre-existing filamentous particles. To test if M2e-specific MAbs affects the morphology of pre-existing filaments, we treated A/Udorn/72 infected cells with M2e-specific MAb 65, 37, 148 or control IgG at 24 h post infection. Interestingly, we found that M2e-specific MAb (65, 37, and 148) caused fragmentation and disruption of the pre-existing filaments (Figs 3b–5a) at 24 h post infection. This can be seen as remnants of filament fragmentation dispersed over the slide (Fig. 4a).

Importantly, treatment with the isotype control IgG, had no effect on the pre-existing filaments (Figs 3b and 5a,b), showing that fragmentation is a specific consequence of M2e-specific IgGs binding to the M2 protein. Thus, M2e-specific MAbs (65, 37, 148) treatment block the filament formation, and cause fragmentation of pre-existing filaments.

## Discussion

Influenza viruses exhibit a range of morphologies from small spherical particles to extremely long filamentous structures. Clinical influenza virus isolates are characterized by the presence of filamentous and even very elongated virions, which can reach microns in length. In addition to influenza, some other respiratory viruses also form filamentous virions, including respiratory syncytial virus and some paramyxoviruses^9,49,50^. For influenza viruses, filament formation is a heritable genetic trait that is selected for in natural transmission. Despite this, long filaments have often been neglected in laboratory studies, and the reason for filament formation remains unclear. The fact that most laboratory strains of influenza present as spherical or kidney shaped virions, the fragility of long filamentous structure as well as the limitations of conventional light microscopy techniques, can explain why filamentous influenza virions are poorly studied. Super-resolution microscopy, a powerful technique that we applied in the present work, allows to visualize and characterize such filaments.

M2e of influenza A viruses is conserved and surface exposed. Although M2e-specific IgG antibodies can protect against influenza A virus challenge in animal model, the *in vitro* growth of most influenza A virus is not affected by M2e-specific antibodies. However, the plaque growth of the influenza virus strain A/Udorn/72 is reduced in the presence of anti-M2 MAb 14C2^24^. In agreement with this, we observed plaque size reduction and reduced replication of A/Udorn/72 in the presence of all three M2e-specific MAbs tested (Fig. 1a–c). A/PR8/H1N1 and A/Udorn/H3N2 virus have a very similar M2 sequence. However, only the A/Udorn/72 strain was sensitive to the *in vitro* inhibition by anti-M2e Mab 65, 37 and 148 (Fig. 1a). The susceptible virus A/Udorn/72 is known to produce filamentous virions^8^. Whether a filamentous virion morphology is a prerequisite for *in vitro* susceptibility to M2e-specific IgG antibodies, remains to be determined. Further, it has been proposed that M2e-specific antibodies could perturb critical interactions between the M1 and M2 proteins, which in turn could affect the interaction of M1 with the vRNP complexes. As a result virion assembly would be compromised, explaining the *in vitro* growth restriction^25^. Antibody-mediated fragmentation of filamentous virions may be due to the induction of a conformation change in the M2 protein, which leads to alterations in membrane curvature^1,8^. M2e-based immunity provides *in vivo* protection against many different influenza A virus strains, most of which are not sensitive to anti-M2e IgG *in vitro*^22,51–54^. The *in vivo* protection by M2e-specific IgG dependent on activating Fcγ receptors that can bind to the Fc portion of IgGs, which in turn leads to phagocytosis or killing of influenza A virus-infected cells^23^. Furthermore, we previously showed that the protection mediated by M2e-specific MAb 65 is dependent on FcγR I and -III receptors^55,56^.

**Figure 6:**
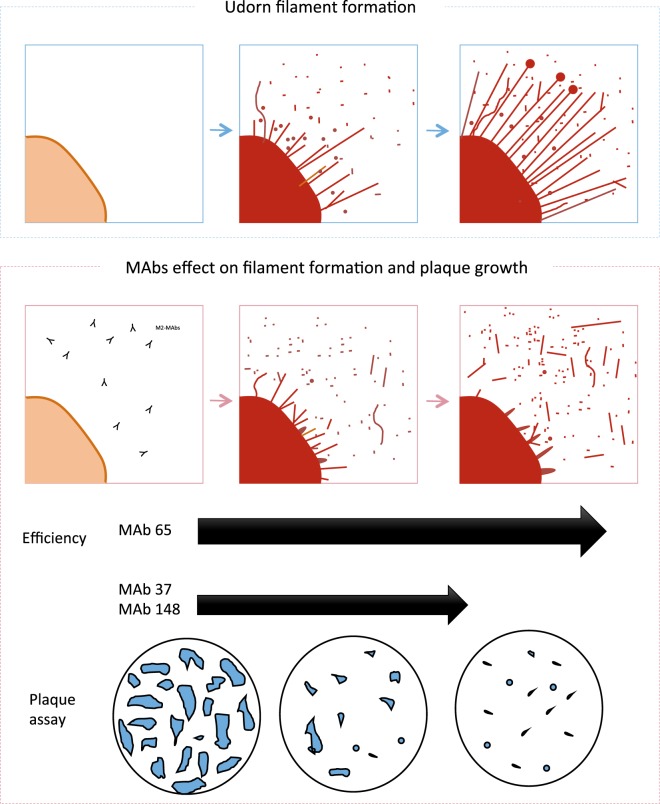
Scheme of the correlation between filament formation and viral plaque formation in influenza A/Udorn/72 virus infected cells. The different M2e-specific MAbs affect the progression of filament formation and plaque growth with variable efficiency in MDCK cells infected with influenza A/Udorn/72 virus.

We used A/Udorn/72 virus because it has been well documented that this is one of the few influenza A virus strains which have retained filament-forming ability after laboratory passage^2,8,10^. The M2 antigen has not been detected previously by immunofluorescence in A/Udorn/72 virus filaments^8^. However, it was presumed that M2 is a viral component of A/Udorn/72 virus filament as the M2-specific antibody 14C2 shown to causes fragmentation of filaments, whereas an M2 inhibitor allows filaments to resist fragmentation at low pH^2,8^. We hypothesized that M2e-specific antibodies can binds to M2 antigen present in filaments structure and causes inhibition of filament formation and fragmentation of preexisting filaments. We decided to address this question using super-resolution STORM microscopy, which allows to trace back individual A/Udorn/72 virus filaments to their base origin on the cell surface. Previously it has been shown that the role of M2 in formation of viral filament is independent of its ion channel activity^8^. M2e immune-stained STORM images acquired at 24 h after infection showed that M2 localizes to the base of budding A/Udorn/72 virus viral filaments (Fig. 2c). Previously, associations were observed between M1 and M2, and between M2 and HA^4^. Co-clustering between M2 and HA suggests the incorporation of M2 at the sites of virus budding, as is indicated in the literature where authors determined that HA and M2 strongly co-cluster in the budozone region of the plasma membrane^30^. The M2e-specific MAb 14C2 previously shown to block the formation of filaments on A/Udorn/72 virus infected cells^8^. Here, we studied how the M2e-specific IgGs treatment of cells inhibited filament formation, and resulted in shorter and wider filaments compared to control IgG treated cells, which showed long and thin filaments with a high expression of HA. The inhibition of filament formation was most apparent in MAb 65 treated cells, which also strongly inhibited plaque growth of A/Udorn/72 virus *in vitro*, and profoundly deformed the shape and width of the filaments (Fig. 6c). The M2e-specific MAbs appeared to blunt filament formation, and resulted in shorter and wider filaments compared to control MAb treated cells (Fig. 6). This decrease is most apparent with MAb 65, which was able to inhibit the formation of filaments. Not only a decrease in size but also a defective structure of filament formation, where filaments does not look thin and long but protrusions with triangle shape. MAb 148 and MAb 37 also inhibited the progression of the filamentous formation but less strongly, producing branched filaments as well. This can be related both to antibody affinity and to the positioning of the epitope on the M2e structure.

Although clinical isolates shown to produce the filamentous phenotype, the functional significance of these diverse filamentous structures remains to be established. It has been suggested that long filaments on the surface of infected cells may be important for cell-to-cell transmission of virus^34,42^. The M2e-specific IgGs may perhaps reduce or prevent cell-to-cell transmission of virus (which would result in a reduced plaque size *in vitro*) through inhibition of filament formation and fragmentation of pre-existing filaments on the surface of infected cells.

In summary, we used confocal and super-resolution STORM imaging technique to characterize A/Udorn/72 virus filaments morphology, studied antibody mediated inhibition of filament formation and fragmentation of pre-existing filaments on the surface of infected cells. Our data demonstrate that M2 is an important player in the formation of filaments on the surface of infected cells. The super-resolution STORM microcopy technique proved to be a valuable tool to study structural details on the plasma membrane or budozone viral components of filaments during IAV replication.

## Material and Methods

### Viruses

Influenza A A/Udorn/307/72 (H3N2) and A/Puerto Rico/8/34 (H1N1) virus strains were amplified on Madin-Darby canine kidney (MDCK) cells in serum-free Dulbecco’s Modified Eagle medium (DMEM) supplemented with non-essential amino acids, 2 mM L-glutamine and 0.4 mM sodium pyruvate) in the presence of 2 μg/mL TPCK-treated trypsin (Sigma) at 37 °C in 5% CO_2_. Ninety six hours after virus inoculation, the culture medium was collected, and cell debris was removed by centrifugation for 10 min at 2,500 g at 4 °C, and the virus was pelleted from the supernatants by overnight centrifugation at 30,000 g at 4 °C. The pellet was resuspended in sterile 20% glycerol in PBS, aliquoted and stored at −80 °C until used. Viral titres in the prepared stocks were determined by plaque forming units on MDCK cells.

### Monoclonal antibodies and polyclonal antibodies and their epitope specificity

The M2e-specific mouse IgG2a MAb 65, IgG1 MAb 37 and MAb 148 have been described (Table 1). Isotype control MAbs directed against the hepatitis B virus core (IgG1) or the small hydrophobic protein of human respiratory syncytial virus (IgG2a) were described^55^. The MAbs were purified from the hybridoma supernatant by protein A sepharose (GE Healthcare). The affinity of MAb 37 MAb 65, and MAb 148 for M2e was determined by ELISA as per method described^55^. Convalescent anti-A/Udorn/72 mouse serum was prepared by infecting BALB/c mice with A/Udorn/72 virus. Briefly, 6–8 weeks old female BALB/c mice were anesthetized by intraperitoneal injection with a mixture of ketamine (10 mg/kg) and xylazine (60 mg/kg) and infected by intranasal administration of 50 μl PBS containing 1 × 10^5^ PFU of A/Udorn/72 virus. Four weeks after infection the mice were anesthetized and then terminally bled. Serum was prepared from the blood, heat inactivated at 56 °C for 30 min and stored at −20 °C. The convalescent mouse serum has high A/Udorn/72 hemagglutination inhibition titers (HAI titer = 1280). All animal experiments were conducted according to the national (Belgian Law 14/08/1986 and 22/12/2003, Belgian Royal Decree 06/04/2010) and European legislation (EU Directives 2010/63/EU, 86/609/EEC). All experiments on mice and animal protocols were approved by the ethics committee of Ghent University (permit number EC2014-074).

### Infection of MDCK cells

MDCK cells were seeded at 3 × 10^3^ cells per well in an 8 well μ-Slide (Cat. No. 80826, ibidi GmbH, Germany) for confocal and super-resolution STORM microscopy or at 3 × 10^5^ in 12 well plates (Corning, USA, REF 353043) for multiple cycle infection. The cells were treated with either MAb 65, 37, 148 or control IgG at concentrations of 20 or 100 μg/mL for 30 minutes prior to mock infection or infection with A/Udorn/72 at MOI 5 for filament analysis. For multiple cycle infection, MDCK cells were infected at MOI 0.01 in serum-free medium containing TPCK-treated trypsin (2 μg/mL, Sigma) and incubated with serum-free medium for 24 h at 37 °C. Twenty four hours after infection, the medium was harvested from 12 well plates and titrated by plaque assay. The cells from microslides were then washed with LPS-free PBS and fixed with 2% paraformaldehyde (PFA) at room temperature for 20 min. The cells were blocked with 1% bovine serum albumin (BSA) solution in PBS for 1 hour at room temperature and stained at room temperature for 1 hour with 1/500 diluted convalescent mouse serum against A/Udorn/72. An Alexa Fluor 647 Donkey anti-Mouse IgG (1/600; Invitrogen) was used as fluorescently labeled secondary antibody. The samples were visualized using a Leica TCS SP5 II confocal microscope (Leica Microsystems, Germany) or Zeiss LSM 780 (Carl Zeiss, Germany) with 40x magnification. Images were analyzed by using ImageJ software. For membrane staining with wheat germ agglutinin (WGA), MDCK cells were grown on Ibidi labtecks at a confluence of 60%. After 24 hours, the cells were fixed with 4% paraformaldehyde for 15 minutes and then washed with PBS. After fixation the cells were stained with WGA-568 (Vector laboratories) at 0.4 µg/mL in PBS for 10 minutes. Subsequently, the cells were visualized under the super-resolution microscope.

### Plaque and plaque size reduction assays

Confluent monolayers of MDCK cells in 12 well plates were infected with 20–50 plaque forming units (PFU) of virus for 1 h at 37 °C. The cells were washed thoroughly and overlaid with 1.2% of Avicel RC-591 (FMC Biopolymer) alone or with MAb 65, MAb 37, MAb 148 or isotype control (IgG1 + IgG2a) at 100 μg/mL supplemented with 2 μg/mL of TPCK-treated trypsin (Sigma). The cells were then incubated for 72 h at 37 °C in 5% CO_2_. Avicel was subsequently removed and the cells were fixed with 4% PFA for 15 min. After permeabilization (10x Permeabilization buffer diluted in bi-distilled water, eBioscience), the cells were stained with 1/2000 diluted polyclonal goat anti-influenza ribonucleoprotein (RNP) (Biodefense and Emerging Infections Resources Repository, NIAID, NIH, NR-4282) followed by donkey-anti-goat IgG HRP-linked antibody (Santa Cruz Biotechnology, cat no. SC2020). After washing, TrueBlue peroxidase substrate (KPL) was used to visualize the plaques. The wells were also scanned and the plaque size was determined using ImageJ Analysis Software.

### Super-resolution optical imaging

Immuno-stained samples were used to acquire STORM images. Cells that had been fixed and stained in ibidi labtech slides were washed once with PBS, after which 300 µl of STORM buffer was added. The STORM buffer consists of an oxygen scavenging system (0.5 mg/ml glucose oxidase, 40 µg/ml Catalase), 5% w/v glucose and cysteamine 100 mM in PBS.

Images were acquired using a Nikon N-STORM 4.0 system configured for total internal reflection fluorescence imaging. Excitation inclination was tuned to adjust focus and to maximize the signal-to-noise ratio. Alexa-647 and WGA-568 fluorophores were excited by illuminating the sample with a 647 nm (160 mW) and 561 (80 mW) laser, respectively, built into the microscope. During acquisition the integration time was 10 ms. For the measurements with Alexa-647 20,000 frames were acquired in the 647 channel. The total time required to acquire one image was about 5 min. For the measurements with WGA-568 40,000 frames were acquired in the 561 channel. The total time required to acquire one image was about 10 minutes.

Fluorescence was collected by means of a Nikon x100, 1.4 NA oil immersion objective and passed through a quad-band-pass dichroic filter (97335 Nikon). Images were recorded onto a 256 × 256 pixel region (pixel size 160 nm) of a sCMOS camera (Hamamatsu). Single-molecule localization sequences were analysed with the STORM plug-in of NIS element Nikon software. Structured illumination microscopy was performed using a Zeiss ELYRA system. For membrane staining of non-infected control MDCK cells, the cells were grown on Ibidi labteck slides at a confluence of 80%. After 24 hours, the cells were fixed with 4% paraformaldehyde for 15 minutes and then washed 3 times for 5 minutes with PBS. After fixation, the cells were stained with WGA-568 (Vector laboratories) at 0,4 µg/ml in PBS for 10 minutes. Then the cells were washed 3 times for 5 minutes with PBS.

### Statistical analyses

Data were analyzed using GraphPad Prism version 7 for Windows(GraphPad Software, San Diego California; www.graphpad.com). The results are shown as individual data points with the mean ± SEM. Statistical analysis of the differences in viral titers, plaque size and *in vitro* viral kinetics were performed using the One-way ANOVA with multiple comparisons correction (Kruskal–Wallis test). For confocal image analysis, the ratio of the perimeter to the surface of cells was determined with the Volocity imaging software (Perkin Elmer). Thresh-holding in the far red channel allows to identify the cells in 3D as objects. For each object the perimeter and surface were measured and extracted from the software. For the quantification of filaments by super resolution microscopy, the acquired images were analyzed using the software ImageJ. We created the following criterion for selecting the filaments to be counted: filaments should have the same width along the entire structure, not be broken or branched. 60 filaments per condition were quantified, with an average of 8 filaments per cell. 43,4% of filaments of each cell did not pass the criterion of selection. The length and width of the filaments were count manually using the ImageJ software. The length was measured after selecting the corresponding scale (0.16 µm/pixel) and the width was obtained by plotting the profile of the filament in ImageJ. Measurements were plotted as dot plot graphics using Graphpad prism software. A value of p ≤ 0.05 was considered statistically significant. The following statistical values and symbols are used through-out the manuscript; non-significant (ns) p > 0.05, *p ≤ 0.05, **p ≤ 0.01, ***p ≤ 0.001; ****p ≤ 0.0001.

## Supplementary information

Supplementary Info
