# Supplementary material for: Super-resolution microscopy reveals significant impact of M2e-specific monoclonal antibodies on influenza A virus filament formation at the host cell surface
Source: Sci Rep. 2019 Mar 14;9:4450. doi: 10.1038/s41598-019-41023-5 (PMC6418112; doi:10.1038/s41598-019-41023-5)
Supplement: Supplementary file 1 — Supplementary Info [file 41598_2019_41023_MOESM1_ESM.docx]

**Super-resolution microscopy reveals significant impact of M2e-specific monoclonal antibodies on influenza A virus filament formation at the host cell surface**

Annasaheb Kolpe^1,2^**^*^**, Maria Arista-Romero^3^**^*^**, Bert Schepens^1,2^, Silvia Pujals^3^, Xavier Saelens^1,2#^, and Lorenzo Albertazzi^3,4#^

^1^ VIB Center for Medical Biotechnology, Technologiepark 927, Ghent, B-9052, Belgium

^2^ Department of Biomedical Molecular Biology, Ghent University, Ghent, B-9052, Belgium

^3^Nanoscopy for Nanomedicine Group, Institute for Bioengineering of Catalonia (IBEC), C\ Baldiri Reixac 15-21, Helix Building, 08028 Barcelona, Spain.

^4^Department of Biomedical Engineering, Institute for Complex Molecular Systems (ICMS), Eindhoven University of Technology, 5612AZ Eindhoven, The Netherlands

***these authors contributed equally to this work**

**#**Corresponding author. Mailing address:

E-mail: [lalbertazzi@ibecbarcelona.eu](mailto:lalbertazzi@ibecbarcelona.eu) xavier.saelens@vib-ugent.be


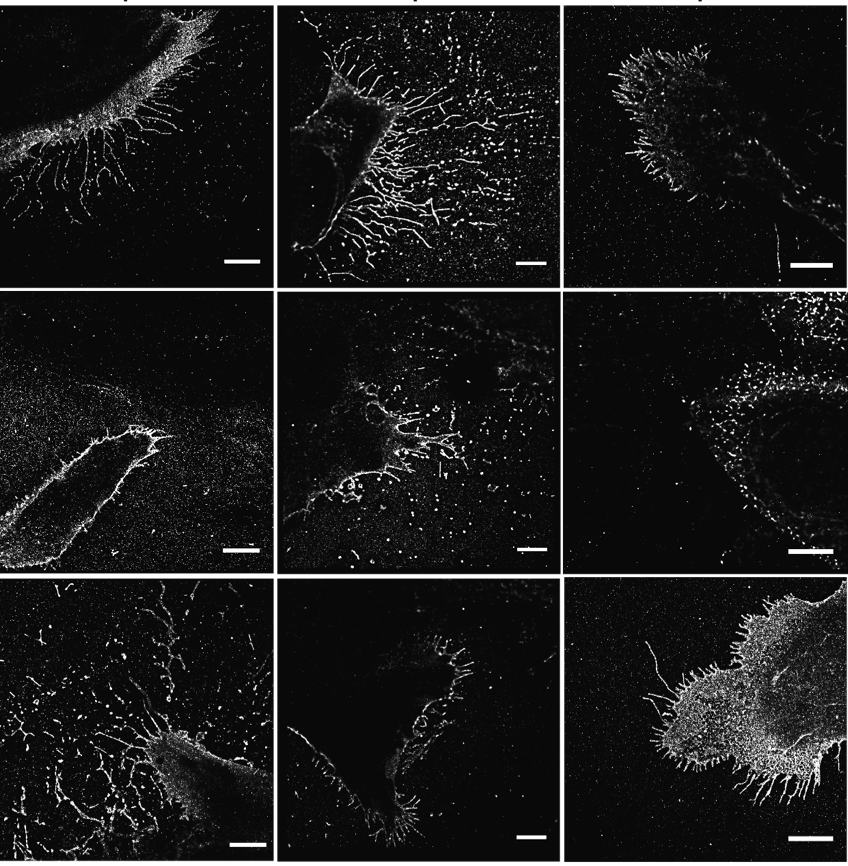


**Supplement figure 1**

90 pfu

30 pfu

Control IgG

MAb 65

MAb 37

300 pfu

**Supplementary figure 1: Super-resolution images a showing M2e-specific IgGs inhibit filament formation in multicycle growth setup.**

MDCK cells (30,000 cells/well) were seeded in 8 well microslides, treated with M2e-speficic MAb 37 (IgG1), MAb 65 (IgG2a), MAb 148 (IgG1), or isotype control IgG1 + IgG2a at 100 μg/mL and then infected with A/Udorn/72 at 300, 90, 30 pfus in TPCK-treated trypsin (Sigma) containing medium and incubated for 24 Hrs at 37°C in serum-free medium. The cells were then washed with PBS and fixed with 2% PFA at room temperature for 20 min. infected cells and A/Udorn/72 filaments were visualized by immune-staining with polyclonal convalescent mouse serum directed against A/Udorn/72, followed by Alexa Fluor 647 Donkey Anti-Mouse IgG serum. Imaging was performed by STORM microscopy. The scale bar represents 5 µm.

**Supplement figure 2**

a b

**c d**

e

**Supplementary figure 2: Distribution of lengths and diameter of MAb 37, 65 and 148**. a) Frequency of lengths of the filaments quantified (n=60) in all cells treated with isotype control (7 cells). b) Frequency of lengths of the filaments quantified (n=60) in all cells treated with 2 different concentrations of MAb 37 (20 μg/mL 8 cells and 100 μg/mL 8 cells). c) Frequency of lengths of the filaments quantified (n=60) in all cells treated with 2 different concentrations of MAb 65 (20 μg/mL 7 cells, 100 μg/mL 7 cells). d) Frequency of lengths of the filaments quantified (n=60) in all cells treated with 2 different concentrations of MAb 148 (20 μg/mL 7 cells, 100 μg/mL 7 cells). e) Frequency of diameters of the filaments quantified (n=60) in cells treated with MAb 37 20 μg/mL; MAb 65 20 μg/mL and MAb 65 100 μg/mL.

**Supplementary table 1**


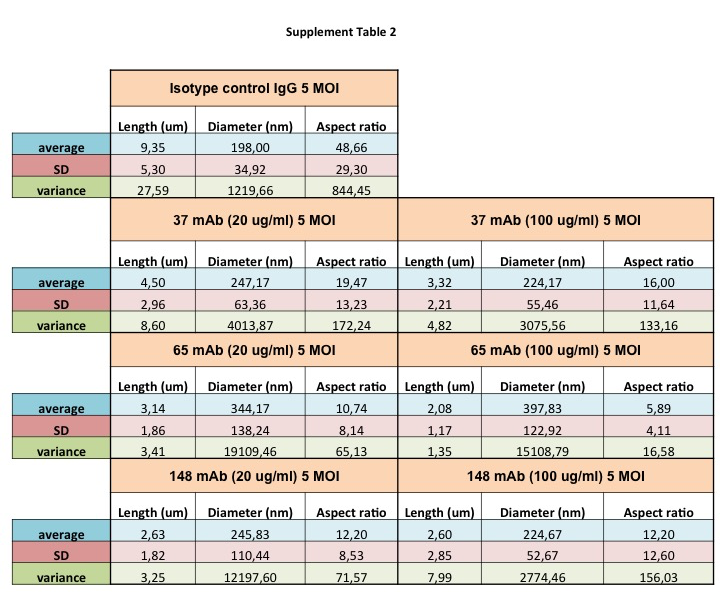


**Supplementary table 1: Measurement of average, standard deviation and variance of all filaments measured:** filaments (n=60) were quantified per condition, measuring length (μm), diameter (nm) and aspect ratio (length/diameter).
